# 385. SARS-CoV-2 Infections Among Military Personnel Deployed on the USNS COMFORT to New York City During the COVID-19 Pandemic and One-Year Follow-Up

**DOI:** 10.1093/ofid/ofab466.586

**Published:** 2021-12-04

**Authors:** Elizabeth Cooper, Tida Lee, Eric Laing, Andrew Ritter, Melissa Lee, Matthew Baker, Tyler Baldino, Terrance Mcadoo, Huy Nguyen, Christopher C Broder, Nusrat J Epsi, Stephanie A Richard, Tyler Warkentien, Eugene Millar, Timothy Burgess, Karl Kronmann, Tahaniyat Lalani

**Affiliations:** 1 Naval Medical Center Portsmouth, Portsmouth, Virginia; 2 Uniformed Services University of the Health Sciences, Bethesda, Maryland; 3 HJF, Bethesda, Maryland; 4 Infectious Disease Clinical Research Program, Department of Preventive Medicine and Biostatistics, Uniformed Services University of the Health Sciences, Bethesda, MD and Henry M. Jackson Foundation, Bethesda, MD, Bethesda, Maryland; 5 Naval Medical Center Portsmouth, Portsmouth, VA, Portsmouth, VA; 6 Infectious Disease Clinical Research Program, Bethesda, Maryland; 7 Naval Medical Center, Portsmouth, Virginia; 8 Infectious Disease Clinical Research Program, Bethesda, MD, The Henry M. Jackson Foundation, Bethesda, MD, and Naval Medical Center Portsmouth, VA, Portsmouth, Virginia

## Abstract

**Background:**

The USNS COMFORT deployed to New York City to augment the inpatient health care capacity in March 2020. The aim of this study was to determine the prevalence of severe acute respiratory syndrome coronavirus-2 (SARS-CoV-2) infection among US Navy personnel upon return from deployment, and to identify incident cases of SARS-CoV-2 infection during 1 year of follow-up.

**Methods:**

Crewmembers, the majority of whom were health care workers (HCW), were enrolled following deployment, in May 2020. PCR results from symptomatic crewmembers during deployment, and Day 0 and Day 14 post-deployment screening swabs conducted on all crewmembers, per military order, were abstracted. A questionnaire and serum were collected on Day 14 post-deployment. SARS-CoV-2 infection was defined as a positive SARS-CoV-2 spike glycoprotein immunoglobulin G antibody (IgG) or PCR. COVID-19 related medical encounters, PCR and antibody testing results within 1 year following deployment were abstracted from the Military Health System Data Repository (MDR). There was adequate provision of personal protective equipment (PPE) in the hospital and the COVID-19 vaccine roll-out for HCW began in December 2020.

**Results:**

Of the 1200 crewmembers, 449 were enrolled and completed the questionnaire and screening swabs, and 432 (96.2%) completed the Day 14 blood draw (Table 1). The cumulative prevalence of SARS-CoV-2 infection was 3.01% (13/432; 95% CI, 1.61%–5.09%). One of 17 subjects did not complete the blood draw and was PCR positive on Day 14. 433/449 (96.4%) had a PCR performed during the follow-up period (i.e. after the Day 14 post-deployment visit until Feb 2021), for HCW screening or symptomatic illness (median number of tests: 2 [IQR: 1, 2; range: 1,6]). 25 of 433 (5.8%) were PCR positive (Fig 1). 19 (76.0%) occurred in corpsmen, 23 (92.0%) were symptomatic and none were hospitalized. One asymptomatic re-infection occurred in a crewmember who was PCR negative and IgG positive at Day 14 post-deployment.

Table 1. Characteristics of the overall cohort and by SARS-CoV-2 infection

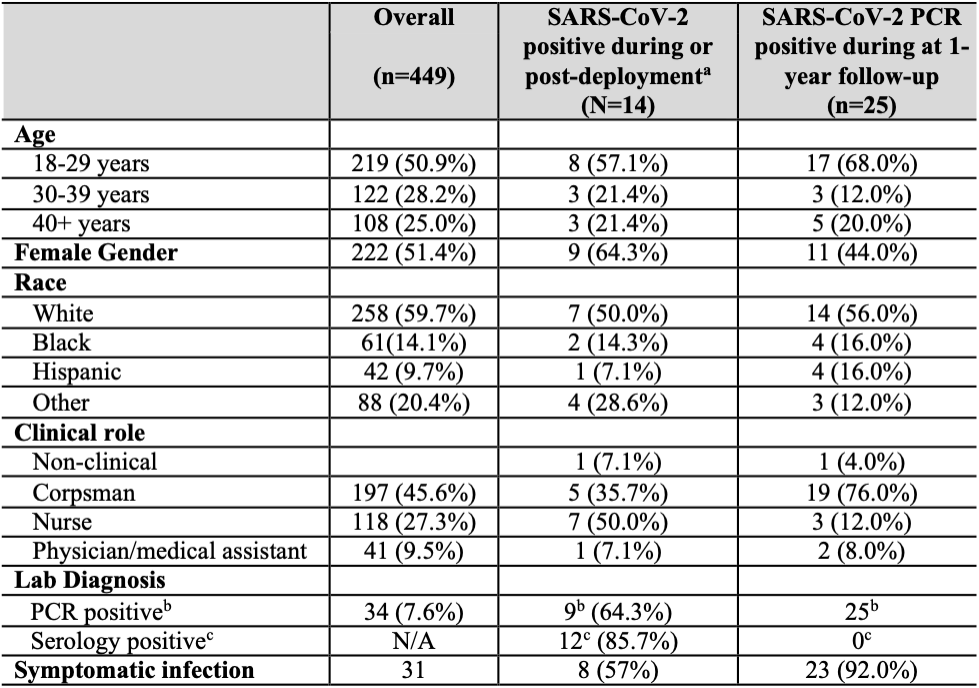

Figure 1. Number of PCR tests (bar graph) and positivity rate (red line) by month in 449 USNS COMFORT crewmembers during 1-year follow-up after return from deployment

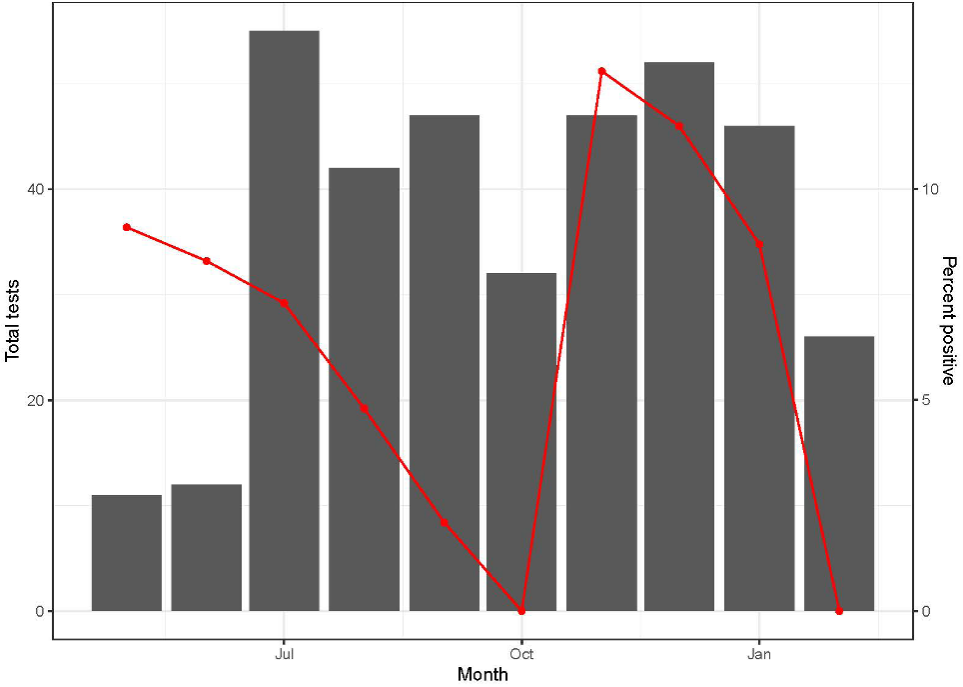

**Conclusion:**

The post-deployment prevalence of SARS-CoV-2 infection was low. A high proportion of HCW underwent PCR testing during 1-year follow-up but a low incidence of infection was observed. This was likely from community transmission as nosocomial transmission was mitigated by adequate PPE and vaccine roll-out.

**Disclosures:**

**All Authors**: No reported disclosures

